# New Jersey State Dental Society

**Published:** 1887-10

**Authors:** 


### NEW JERSEY STATE DENTAL SOCIETY.

BY "FREE LANCE."*

When I started for the New Jersey Society I intended to write you quite fully concerning the meeting, for, as a Jerseyman born and bred, my heart was with them in everything. I found myself handicapped, however, because of their rule of retaining all essays for their own publication, not allowing the appearance of any papers, or the discussions thereon, in advance.

This rule seems to me very unwise, because, as the society publishes only once in three years, many things of importance at the time become stale, or lose their value by reason of the intrusion of fresher matter. However, in the light of the position this society occupies, and the amount of good work it has done, one ought not to quarrel with the methods that have given it success.

I did not pay enough attention to the work of each session to be able to give a thoroughly comprehensive resumé, anticipating access to the stenographer's report. But being barred out by reason of the rule mentioned, I will give you a few details only.

This was the seventeenth annual meeting, held at its regular time, the third Wednesday in July, and, as has been the custom for the last seven years, at the Coleman House, Asbury Park.

Early in the history of the society it traveled from place to place. Becoming older and more sedate, it made Leland's Ocean House, at Long Branch, its headquarters, until 1880, when it moved to Asbury Park. Nowhere on the Jersey coast can be found a room so well adapted to the various needs of a dental society as the ball-room in the Coleman House annex. It is on the second floor, occupying the entire floor, and as the building stands alone, connected only by a covered walk with the hotel, there is light on all sides. One can see at a glance the adaptability of such a room for clinics, exhibits, etc., etc.

The attendance this year, perhaps, was not as large as usual, as far as our own membership was concerned. This was to be expected,

* This letter was unavoidably crowded out of the last number.—EDITOR.

because of the extra attractions of the year in the way of dental meetings. The exhibits, however, were larger and better than ever. This was due, in the main, to the efforts made by Dr. Wm. P. Richards, who, as head of the Committee on Exhibits for some two or three years past, has made a decided success of that department.

The clinics were good, emphatically so, particularly those of Dr. C. C. Carroll, of Meadville, Pa.; Dr. Geo. Evans, of New York City; and Dr. H. A. Parr, of New York City. Dr. Evans was very thorough in all his work, showing how careful he was in the beginning to have the foundation perfect. He took great pains to have his interested listeners follow every step. His device for carrying heat to the extremities of the roots and insuring absolute dryness is very simple, but apparently very effective.

Dr. Parr, as is his custom, performed his work, crowning, from beginning to end, in a very rapid and artistic manner.

Others who gave clinics did equally as well, but I have no notes of their work.

Dr. Frank Abbott, and others, read papers on treatment of deciduous teeth, which elicited free and full discussion. Undoubtedly, in due time, an extended report may be made of these.

Dr. B. F. Luckey, of Paterson, read a very good paper on "Treatment of Abscesses, etc." The discussion thereon was prolonged, and brought out some very diverse views. While the main features of treatment were the same, the drugs to be used, the time to be occupied, and the material to fill with were matters of great difference. Dr. Abbott took exception to the drug used by Dr. Luckey. He argued strongly against iodoform.

Dr. Geo. Evans claimed that by his instrument he succeeded in getting the roots of the teeth so dry, that the iodoform was absorbed and the disinfection complete.

Dr. Abbott was positive in his assertion that no one, by Dr. Evans' method or otherwise, could get the tooth absolutely dry, and that the iodoform lost its power after a time. As I have said, the most opposite views were entertained, and nothing particularly new developed, but in it all the utmost courtesy and good feeling prevailed, and the discussion showed that the minds of many were thinking and studying after the *best* in all things.

Other papers were discussed in much the same manner and spirit of friendliness.

Dr. A. R. Eaton, as President, read a brief but incisive address, which contained some valuable suggestions.

The matter of contributions to the fund started by the First Judicial District Society in regard to the Bridge Patents came up in due time, and the Jersey dentists, with characteristic promptness, walked up to the secretary's desk and put down, not their names merely, but the cash therewith.

G. Carlton Brown, of Elizabeth, was made President, and Dr. Henry A. Hull, Vice-President. The Secretary, Meeker, and Treasurer, Geo. C. Brown, were re-elected as a matter of course.

Before I close this letter I want to ask if you know much of what are called "Pocket Clinics ?"

Judging from what I saw and heard at Asbury Park, those who give such clinics were abroad quite numerously. If a gentleman agrees to clinic, has his name printed and sent broadcast to the profession, if able to be present, should he not do the best he could ? If things are not in condition to enable him to complete the work, ought he not say so, and go as far as able with it, giving his audience the benefit of what instruction he can ?

Some of those who were on the list performed all they had promised from beginning to end, and then took up other work, carrying it as far as time would allow.

Others started their work, and finding themselves unable to finish, explained what they had done to those in attendance, and gave the reason for their inability to complete the operation. Still others, without explanation, refused to clinic, but tried to make appointments at their own offices with the patients obtained for them by others, and failing in this, walked off home. I have seen quite enough of this for a life-time.

There were present at the meeting the usual types. We had the dentist who has carefully announced in his local papers that Dr. So-and-So was in attendance upon the State Dental Society, but who put in all his time at the Long Branch race-course.

Then we had the genuine student, who came there to learn, and who by no amount of coaxing could be induced to take up the role of teacher, though sometimes well qualified for the work. Then there was the man who is always on his feet, no matter what the subject in hand. But I need not sum them up. You have seen them all, for they are present at every dental meeting.

